# Doxorubicin increases permeability of murine small intestinal epithelium and cultured T84 monolayers

**DOI:** 10.1038/s41598-020-78473-1

**Published:** 2020-12-08

**Authors:** Paul Cray, Breanna J. Sheahan, Jocsa E. Cortes, Christopher M. Dekaney

**Affiliations:** Department of Molecular Biomedical Sciences, College of Veterinary Medicine, NC State University, 1060 William Moore Drive, Campus, Box 8401, Raleigh, NC 27607 USA

**Keywords:** Cell biology, Physiology, Gastroenterology

## Abstract

Enteric bacteria and/or their products are necessary for doxorubicin (DXR)-induced small intestine mucosal damage. While DXR does not induce gross loss of epithelium, others have shown elevated serum endotoxin after DXR administration. However, the mechanism of movement is unknown. We hypothesized that DXR treatment resulted in increased paracellular translocation of bacteria or bacterial products through the small intestinal epithelium. We measured permeability after DXR administration using transepithelial resistance and macromolecular flux and assessed tight junctional gene expression and protein localization both in vitro using T84 cells and ex vivo using murine jejunum. DXR treatment increased flux of 4 kDa dextrans in mouse jejenum, but increased flux of 4, 10 and 20 kDa dextrans in T84 cells. Following DXR, we observed increased permeability, both in vitro and ex vivo, independent of bacteria. DXR induced increased expression of *Cldn2* and *Cldn4* in murine small intestine but increased only *CLDN2* expression in T84 cells. DXR treatment induced disorganization of tight junctional proteins. We conclude that DXR increases paracellular transit of small macromolecules, including bacterial products, through the epithelium, by altering expression of tight junctional components and dynamic loosening of cellular tight junctions.

## Introduction

Mucositis of the intestinal tract is a common side effect associated with clinical use of chemotherapy and irradiation. Our lab previously demonstrated that doxorubicin (DXR), a common chemotherapeutic, rapidly induces apoptosis in the crypt epithelium of the murine small intestine^1^. This leads to mucosal damage characterized by impaired epithelial proliferation, loss of crypts, and villus blunting^1^. Concomitantly, we observed significant increases in chemokine expression and infiltration of macrophages and neutrophils into the lamina propria space surrounding crypts^2^.

We subsequently demonstrated that this mucosal damage and innate immune response is dependent on the presence of enteric bacteria by comparing conventionally raised mice to germ free mice and mice treated with oral antibiotics^2,3^. While the initial apoptosis still occurred in the epithelium of germ free and antibiotic-treated mice after DXR, the subsequent chemokine production, immune cell infiltration, and mucosal damage were prevented^2,3^. Others have demonstrated that prevention of signaling (e.g. via whole body knockout or antagonists) through toll-like receptors (TLRs), such as TLR-2, TLR-4, or TLR-9, which are present on the cells of the epithelium and the subepithelium, reduces DXR-induced intestinal damage^4,5^. These studies suggest that bacterial penetration of the epithelial barrier and resultant pathogen-associated molecular pattern (PAMP)-mediated stimulation may play an important role in the bacterial-dependent mucosal damage and immune infiltration observed after DXR.

The epithelium of the intestinal tract functions as a selective barrier. This proliferative single cell layer has to balance the dynamics of nutrient absorption, high cellular turnover, and ion movements, while concurrently preventing PAMP-mediated stimulation of the immune system and translocation of enteric bacteria into the systemic circulation^6,7^. Disruption of any one of these functions can result in acute inflammation or instigate progression into a chronic condition such as ulcerative colitis or Crohn’s disease^7,8^. In the case of DXR treatment, others have reported increased water and albumin movement into the intestinal lumen and the presence of endotoxin (LPS) in the systemic circulation, suggesting that the barrier may be compromised^9,10^.

The epithelial barrier is maintained by the connections between epithelial cells, formed of a series of junctional protein complexes. The tight junction (TJ) is the most important of these protein complexes, as it modulates the interepithelial pore size to allow for ion movement, and is selectively permeable to the movement of macromolecules^7^. The TJ is principally made of a collection of Occludins, junctional adhesion molecules, and Claudins, all of which have different pore forming and restricting functions^7,11^. Together, these molecules make a fluid transcellular structure that maintains a seal around the polarized cells. The TJ is anchored to the cytoskeleton with a series of linker proteins, such as Zona Occludens-1 (ZO-1)^11^. Additionally, these structures segregate epithelial cells into apical and basolateral membranes, which regulates the distribution of membrane-bound receptors. Loss of this segregation function has been implicated in the inappropriate activation and signaling of a proinflammatory response^6,8,12^. However, little is known about the effect of DXR on the composition and integrity of the epithelial tight junctional network.

In this study, we hypothesized that DXR specifically increases small intestinal epithelial permeability, which allows enteric bacteria and/or their PAMPs to transit the mucosal barrier. Our data show that DXR administration, both in vitro and in vivo, alters tight junctional transcripts and protein localization. Functional testing of both intestinal mucosa ex vivo and T84 cells via Ussing chambers and measurement of Transepithelial resistance (TER) demonstrates that DXR exposure induces increased permeability. This increased permeability is sufficient for small molecules, such as bacterial PAMPs, to reach the underlying lamina propria, but not for bacterial translocation. Together, these data suggest that our prior findings demonstrating the importance of enteric bacteria in DXR mucositis are likely due to paracellular movement of bacterial PAMPs, such as LPS, across the barrier, resulting in PAMP-mediated stimulation of TLRs and other receptors.

## Results

### Doxorubicin loosens the tight junctional barrier in vitro

**Figure 1:**
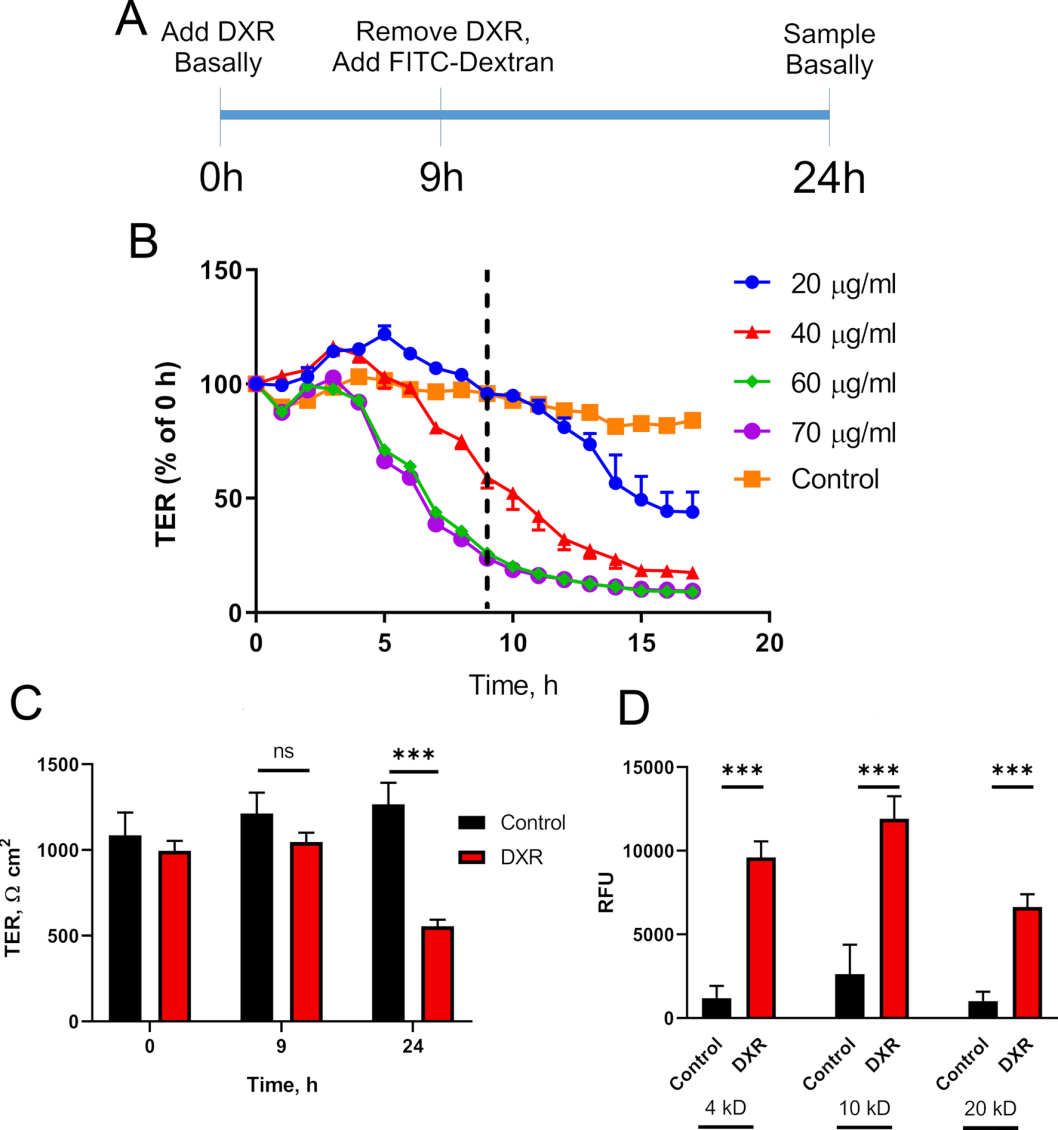
Doxorubicin exposure increases permeability of T84 cells. (**A**) Experimental design for in vitro model exhibiting doxorubicin (DXR; 40 µg/ml) application and FITC-Dextran flux in confluent T84 cell culture. Time after application of DXR is indicated in hours (h). (**B**) Transepithelial resistance (TER) measurements over time after application of various concentrations of DXR. Dashed line indicates time of DXR removal. (**C**) TER measurements over time in control and DXR treated (40 µg/mL) T84 transwells. (**D**) Macromolecular flux measurements of FITC-Dextran molecules of varying size at 24 h post-DXR (40 µg/mL). Statistics: (**A**) Repeated Measures 2 way ANOVA followed by Tukey’s HSD; (**B**) n = 3 wells per concentration. (**C**) Student’s *t*-test; ***p < 0.001; n = 6 wells per treatment. (**D**) Student’s *t*-test; ***p < 0.001; n = 6 wells per each group.

We used an in vitro 2D epithelial monolayer to interrogate the epithelium-specific effect of DXR isolated from other cell types. T84 cells, a human-derived immortalized colon cancer cell line, form a tight, confluent barrier, suitable for use as an in vitro model for assessing barrier function. Loosening of the tight junctional network is signified by a decreased TER, resulting from increased ionic movement via the paracellular space^6,7^. From our previously published in vivo experiments, epithelial DNA damage and apoptosis will have peaked by 9 h post exposure, thus we examined our in vitro model at this time point and at 24 h post DXR (Fig. 1A)^1^. T84 cells respond to DXR in a dose and time dependent manner (Fig. 1B). Based upon our preliminary findings, we used a concentration of 40 µg/mL DXR for the remainder of our experiments as this concentration caused a decrease in TER, but did not result in cell loss as assessed with brightfield, acridine orange/propidium iodide (AO/PI) staining, and TER (Fig. 1B). TER of DXR-treated T84 cells was significantly lower than control cells 24 h after treatment (Fig. 1C).

Next, we assessed macromolecular movement through the monolayer by measuring fluorescently labeled dextran flux^13^. By utilizing fluorescently-labeled dextrans of varying sizes (4, 10, and 20 kDa), we were able to determine what size molecules would be able to transit the pores between epithelial cells. These sizes correspond to components like LPS (20 kDa), principally Lipid A (4 kDa) which is the main PAMP signaling portion of LPS, as well as bacterial DNA and RNA^14,15^. After DXR, there were significant increases in the relative fluorescence of FITC-labeled dextran for 4, 10, and 20 kDa sized molecules in basal media in the DXR treated T84 cells compared to their respective control groups (Fig. 1D). This indicates that T84 cells, in the presence of DXR, undergo loosening of the tight junctional network to an extent that molecules as large as 20 kDa, such as LPS, may access the basolateral region of these cells.

### Doxorubicin exposure causes tight junctional remodeling in vitro

**Figure 2:**
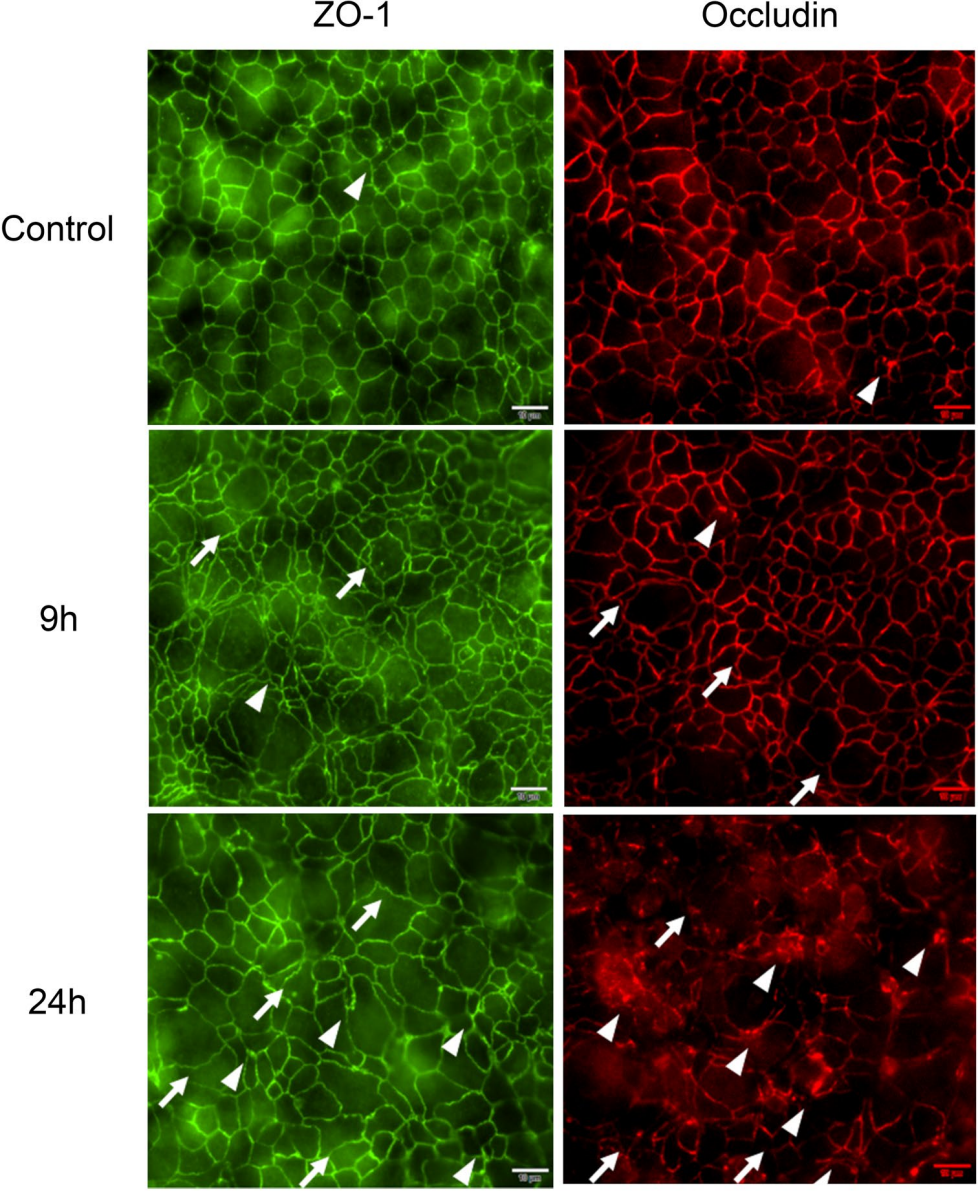
Doxorubicin administration perturbs the tight junctional network in T84 cells. Representative images of immunofluorescent staining of ZO-1 and Occludin after DXR application to T84 cells, *n* = 3 for each time point. Aggregates of ZO-1 and Occludin (arrowheads) suggest an internalization of tight junctions. Uneven pulling of the cytoskeleton on the tight junctional network is observed as sinusoidal irregularities (arrows). Occludin pseudo-colored red for clarity. Scale bar 20 μm.

ZO-1 and Occludin, two major proteins of the tight junctional network, exhibit a classic ‘chicken wire’ appearance between cells in normal epithelial tissue (Fig. 2, Control). DXR treatment of T84 cells caused disorganization of the tight junctional network, indicated by the increased presence of membrane aggregates of each protein (Fig. 2, arrowheads). These aggregates likely represent endocytosed tight junctional proteins that have been vacuolized via cellular recycling processes^16^. Further, DXR treatment caused the cellular membranes to develop an increased sinusoidal phenotype, indicating uneven pulling of the tight junctional network by the cytoskeleton (Fig. 2, arrows)^17^. Membrane disorganization progressively worsened after DXR, and was broadly apparent throughout the monolayer by 24 h. These data suggest that ZO-1 and Occludin are internalized and degraded rapidly after DXR.

### Doxorubicin exposure modulates in vitro tight junctional gene expression

**Figure 3:**
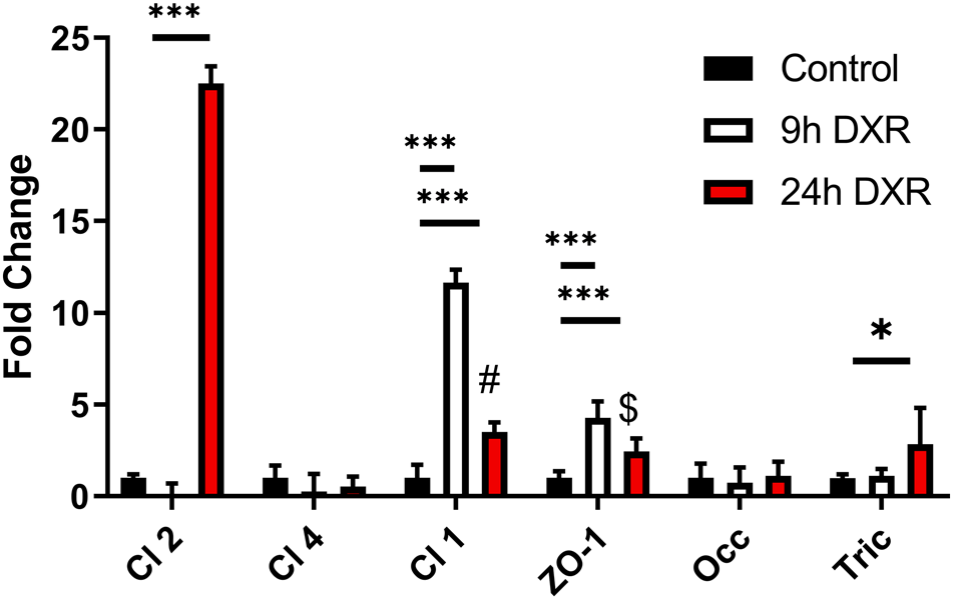
Doxorubicin exposure modulates tight junctional gene expression in a time dependent manner. RT-qPCR of T84 cell mRNA at 9 h and 24 h after DXR exposure with time respective controls. The experimental design is the same as Fig. 1A. All data is presented as fold change normalized to *ACTB*. n = 6 wells/ group. Statistics: 2 way ANOVA followed by Tukey’s HSD; ***p < 0.001.

Our TER and flux data suggest that DXR treatment disrupts normal barrier function of the intestinal epithelial tight junctions. To understand the transcriptional response to DXR in the in vitro model, we performed gene expression of characteristic tight junctional genes *Claudin1* (*CLDN1*), *Claudin2* (*CLDN2*), *Claudin4* (*CLDN4*), *Zona Occludens1* (*ZO1*), *Occludin* (*OCC*), and *Tricellulin* (*TRIC*) (Fig. 3). No significant transcriptional changes to *OCC* and *CLDN4* were observed 24 h after DXR treatment (Fig. 3). There were significant increases in expression of *CLDN1* and *ZO1* by 9 h post DXR, suggesting the cells responded to the DXR by upregulating junctional proteins responsible for tightening the epithelial barrier (Fig. 3). *CLDN2*, in contrast, was significantly upregulated only at 24 h post-DXR (Fig. 3). *CLDN2* is associated with so called ‘leaky’ tight junctions, where increased expression is indicative of enhanced barrier permeability to cations and water and can impede formation of sealing tight junctions^6,18^. Upregulation of *CLDN2* concomitant with *CLDN1* and *ZO1* decline suggests that following DXR exposure the cells shift to a transcriptional response representative of a leaky tight junctional network, which is supported by the previously observed decrease in TER (Fig. 1C) and increased movement of macromolecules (Fig. 1D)^7^. We observed an increase in *Tricellulin *(*Tric*), one of the main regulators of macromolecular flux 24 h after DXR (Fig. 3). Loss of normal tight junctional complexes in our in vitro model was suggestive of decreased barrier function and increased permeability, thus we next investigated barrier function after DXR exposure in vivo^8^.

### Doxorubicin lowers transepithelial resistance ex vivo independent of bacteria

**Figure 4:**
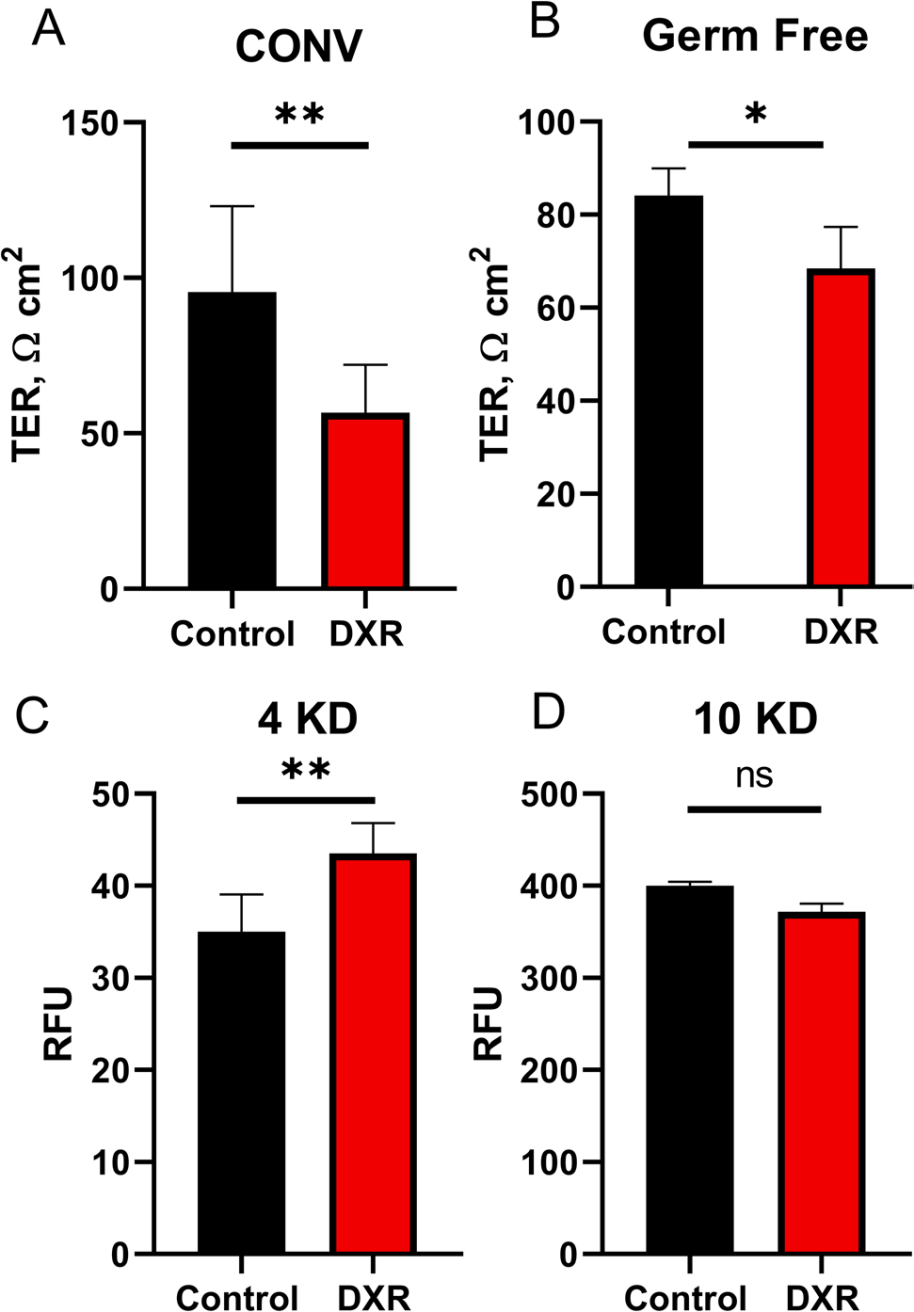
Doxorubicin in vivo increases permeability to small molecules in mouse jejunum. (**A**) TER measurements of conventionally raised (CONV) animals (*n* = 3/group). (**B**) TER measurements of germ free (GF) animals (*n* = 3/group). (**C**,**D**) Macromolecular flux measurements of jejunal tissue isolated from CONV mice (*n* = 4/group). FITC-Dextran molecules of 4 kDa (**C**) and 10 kDa (**D**) were applied to the mucosal side at 24 h post-DXR (40 µg/mL), and the fluorescence of the serosal chamber was measured after flux. Student’s *t*-test; *p < 0.05; **p < 0.01; *ns* no significance.

We utilized Ussing chambers to measure TER and macromolecular flux on isolated murine jejunal tissue from germ free (GF) and conventionally (CONV) raised wild type mice to evaluate whether the presence of bacteria influenced the effect of DXR on barrier permeability. Analysis 24 h after DXR administration demonstrated a decrease in TER in jejunal tissues of both GF and CONV mice, as compared to their non-DXR treated control tissues (Fig. 4A,B). Macromolecular flux using fluorescently labeled dextrans demonstrated that only 4 kDa molecules transited the barrier of CONV mice after DXR, while 10 kDa molecules did not (Fig. 4C,D). We did not assess larger molecular weight molecules. These data demonstrate that that the DXR-induced drop in TER is independent of the presence of luminal bacteria, and that molecules < 10 kDa have enhanced paracellular movement across the epithelial barrier after DXR exposure.

### Doxorubicin does not increase the presence of bacterial DNA in peripheral lymphatic tissues

**Figure 5:**
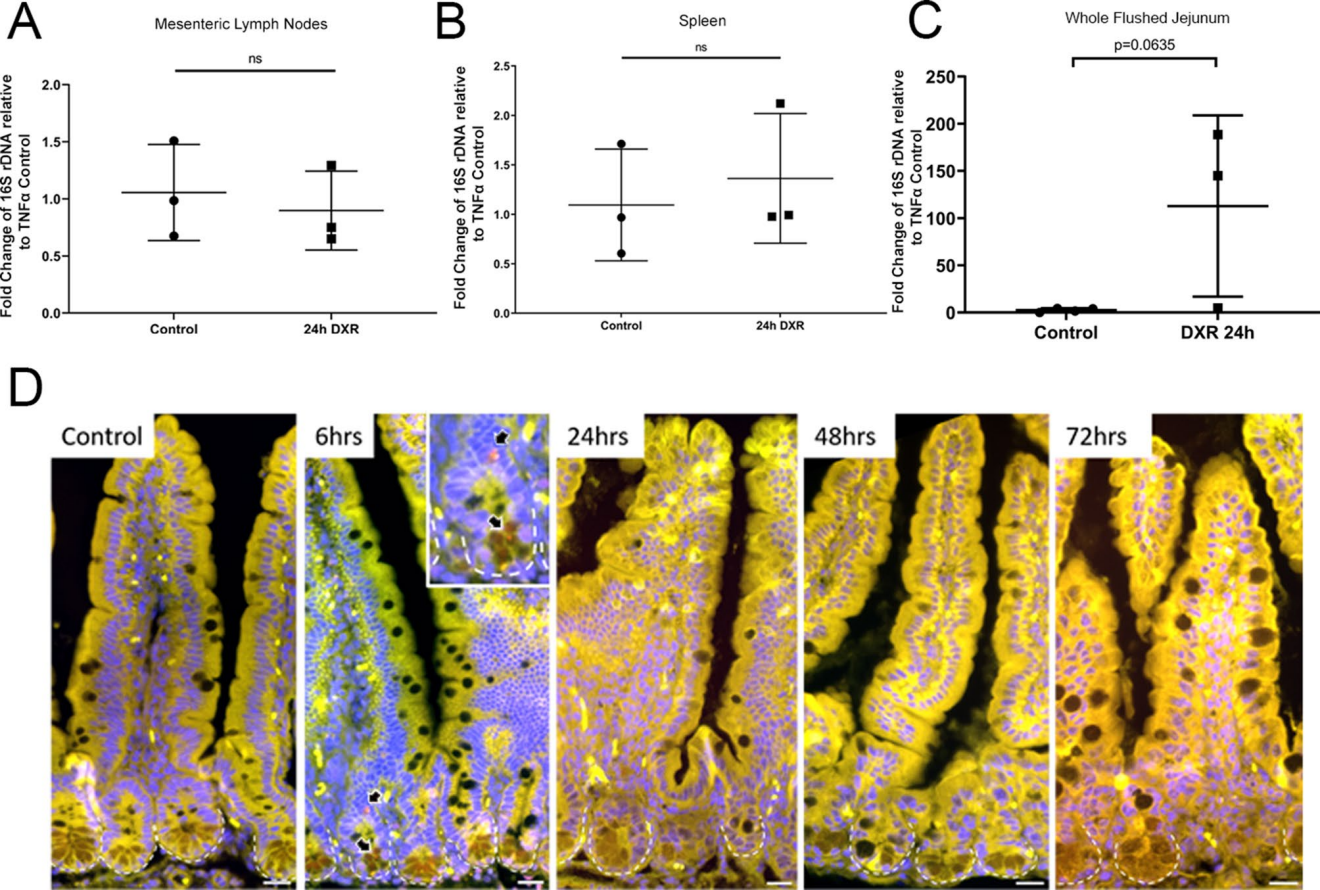
DXR in vivo does not result in bacterial translocation into the local lymph tissues or submucosa. (**A**–**C**) Mesenteric lymph nodes (**A**), spleen (**B**), and whole flushed jejunum (**C**) were isolated from control mice or mice treated with DXR. 16S rDNA quantification was performed relative to host TNFα transcript expression. (**D**) Representative 16S rRNA fluorescence in situ hybridization (FISH) images of flushed jejunal tissue at various time points after doxorubicin injection (6, 24, 48, and 72 h) as compared to control jejunum. Red arrows indicate 16S FISH-positive (red) bacteria in a jejunal crypt from a mouse treated with DXR 6 h prior to sacrifice (inset). Images are representative of *n* = 3 per time point. Scale bar 20 μm. Statistics: (**A**) (n = 3/group), Student’s *t*-test, *ns* no significance.

To investigate whether DXR’s effects on the epithelial barrier led to bacterial translocation into the submucosal tissues and systemic circulation, we quantified bacterial DNA in the whole flushed jejunal tissue, jejunal mesentery, and spleens isolated from CONV mice 24 h after in vivo DXR administration. There were no differences in the quantity of bacterial 16S rDNA in either the mesenteric lymph nodes (Fig. 5A) or the spleen (Fig. 5B) by 24 h after DXR when normalized to TNFα, a single copy gene in host DNA^19,20^. Although there was a trend toward increased bacterial 16S rDNA in whole flushed jejunum, this was non-significant (Fig. 5C). This may be reflective of increased mucoadherent bacterial populations or early bacterial translocation that has not progressed beyond local tissues. Correspondingly, previous work has shown that increases in endotoxin levels are not apparent until 48 h after exposure^10^.

### No increase in translocation of bacteria is observed after doxorubicin exposure

To clarify whether there may be local bacterial translocation occurring after DXR, we performed fluorescence in situ hybridization with a 16S rRNA eubacterial probe or nonsense probe on flushed jejunal tissues from control and DXR treated conventionally raised mice at multiple time points post DXR (Fig. 5D). No bacteria were identified within the crypt lumen in control tissues, consistent with the high concentration of antimicrobial peptides present in the crypt lumen^21^. However, at 6 h after DXR, rare bacteria were present in either the crypt lumen or the apical cytoplasm of crypt cells (Fig. 5D, arrows). It is unknown whether the composition of antimicrobial peptides might be altered after DXR administration, allowing for migration of bacteria down into the crypt lumen. Given that bacterial translocation may occur later than 24 h after DXR administration, we also examined tissues at 48 and 72 h after DXR. We were unable to detect bacteria in the lamina propria in any of the jejunal tissues examined (Fig. 5D). Overall, these data suggest that substantial bacterial translocation is not the cause of the bacterial-dependent damage observed in conventionally raised mice after DXR.

### Doxorubicin induces a pore forming phenotype in murine jejunum

We hypothesized that the bacterial-independent DXR mediated drop in TER and increased macromolecular flux in vivo was due to reorganization of the tight junctional network, as suggested by our in vitro data. Therefore, we evaluated gene expression and cellular localization of several tight junctional proteins in conventionally raised murine jejunal epithelium, including Claudin-2, Claudin-4, and ZO-1. Claudin-4 is a multifunctional claudin, which principally acts as a sealing claudin but has also been shown to decrease Na^+^ permeability while increasing Cl^-^ permeability^22,23^. Thus, altered Claudin-4 presence in the tight junctional network may affect the charge of the typically negatively charged crypt lumen and charge sensitive antimicrobial peptide function^24^. Further, alterations in solute concentrations, particularly chloride, can lead to luminal distension, which has been observed after DXR treatment^9,25,26^.

**Figure 6:**
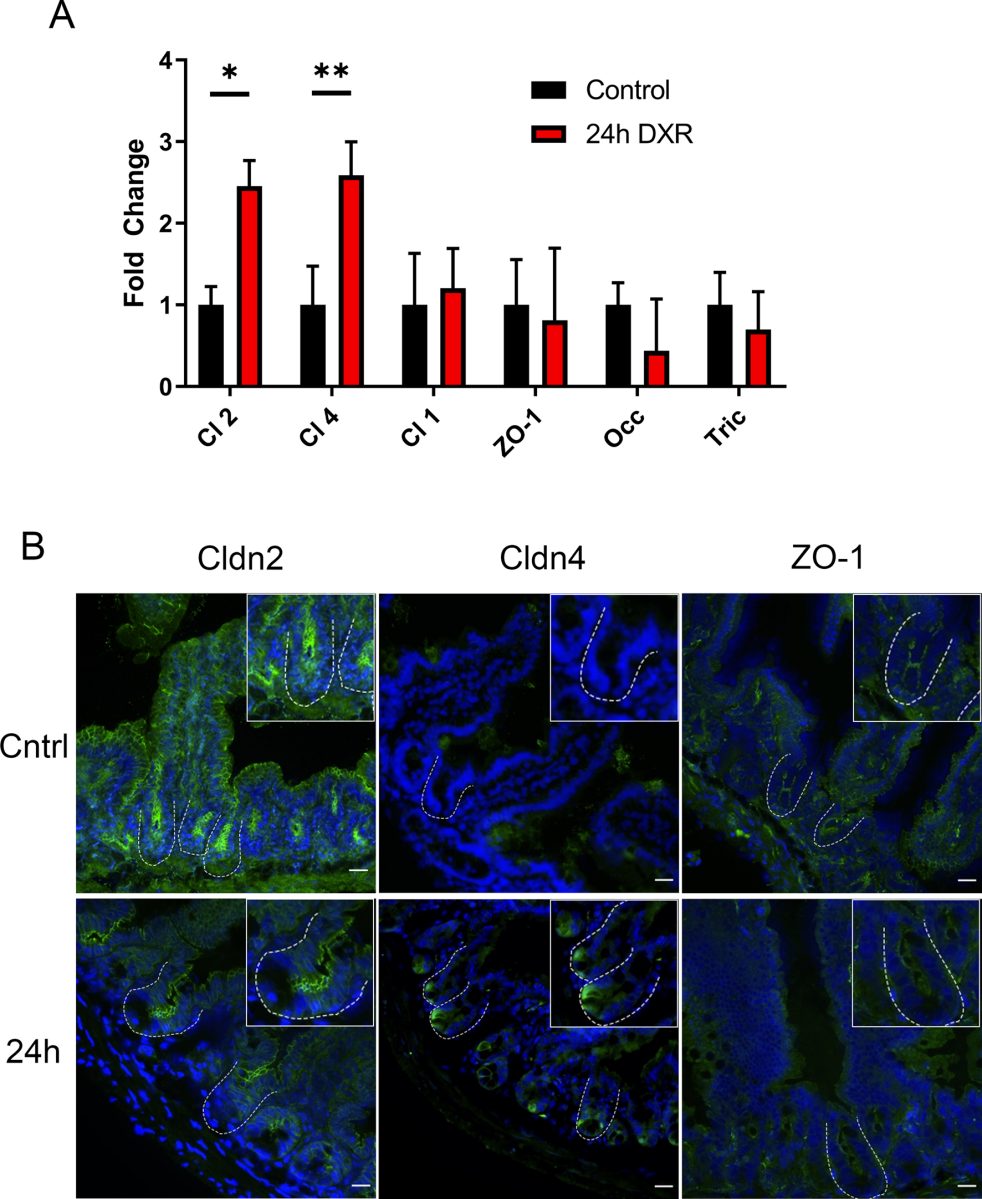
The murine jejunal tight junctional network is affected by DXR exposure. (**A**) RT-qPCR of tight junctional genes in jejunal crypt epithelium isolated from control tissues and tissues collected 24 h after DXR from conventionally raised mice (*n* = 3/group). All data is presented as fold change normalized to *Actb*. Student’s *t* test; *p < 0.05, **p < 0.01. (**B**) Representative images of immunofluorescent staining of tight junction associated proteins Claudin-2 (Cldn2), Claudin-4 (Cldn4), and Zona Occludens-1 (ZO-1) in control tissues (Cntrl) and tissues collected 24 h after DXR (24 h) from conventionally raised mice. Images are representative of *n* = 3 per time point. Scale bar 20 μm.

DXR induced significant transcriptional upregulation of *Cldn2* and *Cldn4* in isolated jejunal crypt epithelial cells, whereas no transcriptional response was observed for *Cldn1*, *ZO-1*, or *Occ* (Fig. 6A). We did observe a non-statistically significant negative trend in *Tricellulin* (Fig. 6A). Claudin-2 continued to exhibit a membrane-bound localization but appeared modestly reduced at the base of DXR-treated jejunal crypts compared to control crypts. However, Claudin-2 distribution and intensity did not appear affected in the majority of the villus epithelium in DXR-treated mice compared to control mice (Fig. 6B). Claudin-4 protein had increased immunofluorescent labeling in cells located at the crypt base after DXR treatment, in contrast to the minimal positivity observed in control crypts (Fig. 6B). After DXR, ZO-1 immunofluorescence was disorganized and faint in jejunal epithelium as compared to the well-organized typical chicken wire pattern observed in control tissues. These data indicate that alterations to the tight junctional network and its cellular localization occur after DXR treatment. Additionally, based on the increased *Cldn2* expression and the enhanced crypt base-specific localization of Claudin-4, we speculate that DXR rapidly shifts the tight junctional network phenotype to a pore forming state.

## Discussion

We have previously shown that enteric bacteria are required for DXR-induced damage to the small intestinal mucosa^2,3^. As other studies have demonstrated that immune activation is important in the pathogenesis of DXR-induced mucositis, our goal here was to identify DXR’s impact on the epithelial barrier^5,10^. In this study, we have demonstrated that DXR treatment increases permeability of the intestinal epithelial barrier to ionic movement and small proinflammatory molecules.

We demonstrated increased barrier permeability following DXR treatment *both *in vitro and in vivo, including in germ free animals. This indicates that the change in barrier integrity is due directly to DXR and not dependent upon the presence of intestinal bacteria. We also demonstrated that this increase in permeability allowed the flux of small molecules, but not bacterial translocation. This is consistent with our damage model that DXR induces DNA damage and crypt-specific apoptosis with no gross epithelial loss^1^. It further suggests that proinflammatory molecules such as Lipid A, the active portion of LPS, likely exhibit increased paracellular flux after damage from DXR, resulting in immune system activation and endotoxemia^10^. Recent work by Huang et al. supports this, showing that 5 days after DXR exposure, serum LPS levels were elevated compared to control but were not significantly increased 2 days after DXR exposure^27^. Our work here suggests that the loosening of the intestinal barrier develops temporally after DXR administration.

We showed no increase in bacteria in immune and local tissues (Fig. 5A–C) which is in contrast to previous findings showing an increase in CFUs present in these tissues at 48 h after DXR^28^. However, our methods were different in that we assessed total bacterial 16S rDNA present in these tissues, irrespective of the bacteria’s ability to be cultured. Also, DXR has been shown to change the microbiota make up and this change may explain why some bacteria were able to be cultured in the other study^27^. Thus, while there may not be an increase in bacterial 16S rDNA present, there may be a shift in the population allowing for bacteria that can be cultured to be present.

The increase in permeability was associated with alterations in gene expression and cellular localization of several tight junction proteins. Our in vitro data show that DXR treatment causes internalization of the tight junctional components ZO-1 and Occludin. Loss of membrane associated tight junctional proteins is indicative of altered barrier function^6^. Additionally, in vivo DXR exposure increased the transcript levels of pore forming *Cldn2*. Further, increased expression of *Cldn2* has been shown to increase the number of TJ strand breaks and an increase in TJ discontinuities, suggesting a more disorganized tight junctional network after DXR^18^. Tricellulin has been shown to shore up TJ discontinuities and the increased expression observed at 24 h may be a compensatory mechanism to mitigate the increased discontinuities from *Cldn2* while also attempting to seal tricellular junctions^29^. This discrepancy between the in vitro and in vivo data for the tricellulin expression may be explained by the persistence of an inflammatory state after DXR in vivo compared to in vitro. Tricellulin has been shown to be decreased in chronic inflammatory states such as IBD, occurring simultaneously with an increase in *Cldn2* expression as well as an increase in 4 kDa macromolecular flux, all of which has been shown to be due to elevated IL-13 produced by resident immune cells^30,31^. By 6 hours in vitro and in vivo, the peak amount of apoptosis after DXR treatment has occurred^1^. However, fulminant crypt loss in vivo due to inflammatory cell infiltration peaks 3 days after DXR^3^. This later time period would not be captured by the in vitro system. Together, these data suggest an opening of the paracellular space between the epithelial cells. The modest reduction in Claudin-2 immunofluorescence in the crypt base may indicate there are spatially-restricted differences along the crypt-villus axis. As the base of the crypt also exhibited increased Claudin-4 immunofluorescence, these data suggest there may be a localized enhancement of the barrier at the crypt base. Previous literature suggests that Claudin-4 functions to tighten the barrier while also allowing for anion selective movement^22,23,32^. Thus, the presence of Claudin-2 expression in the transit-amplifying region of the crypt would suggest an increase in the flow of Na^+^ and subsequently water into the luminal space^32,33^. Since the crypt base houses the intestinal stem cells and Paneth cells, alterations in barrier function might play a role in epithelial regeneration after damage. In the future, single cell transcriptional analysis would clarify whether crypt base cells have a different transcriptional response to DXR in comparison to the rest of the epithelium.

Recent work by Raju et al. has shown that increased expression of Claudin-2 enhances immune mediated colitis specifically via the pore pathway and not via the leak pathway; however, they also showed that *Cldn2*^−/−^ were protected from the immune mediated increase in the leak pathway^34^. The pleiotropic nature of tight junctional proteins, specifically Claudin-2, suggests that changes in expression may have effects on both the leak and pore pathway^35^. While it is still debated whether Claudin-2 can directly affect macromolecular flux, increased expression of Claudin-2 has been implicated in increased movement in both the pore and leak pathways^34,36,37^. Of particular note, Tsai et al., using a bacterial infection model, show first an increase in the pore pathway permeability, which was indicated by the increase in *Cldn2* expression, followed by an increase in the leak pathway^37^. Additionally they show, increases in the pore pathway may lead to further increases in the leak pathway^37^. While our work here has shown largely changes to the pore pathway, our modest increase in macromolecular flux suggests that changes to the leak pathway have occurred as well. Indeed, increases in bacterial endotoxins by 5 days further suggest that the leak pathway permeability is enhanced after DXR exposure^10,27^.

In conclusion, DXR exposure initially causes a shift to a leaky, pore forming transcriptional response with spatially dependent changes along the crypt-villus axis suggesting that there are crypt and villus specific responses to DXR. This is then followed by an increase in the leak pathway causing the increased movement of bacterial products across the barrier. This may be associated with the sensitivity of the crypt epithelium to damaging agents such as chemotherapeutics and irradiation^1^. Further, this deviation in the tight junctional network combined with an increase macromolecular flux of 4 kDa suggests that the barrier is dynamically loosening. In light of this, strengthening the epithelial barrier with pretreatments, such as PGE2 or *Bifidobacterium bifidum* may prevent damage induced by DXR and other agents^38,39^. Additionally, a recent study demonstrated that Claudin-7 is critical for stem cell self-renewal in homeostasis^40^. Further studies are necessary to determine if the epithelial junctional network can be manipulated to mitigate the bacterial-dependent component of DXR-induced mucositis or to enhance stem cell regeneration after epithelial damage as well as further resolve the changes to the barrier over time.

## Methods

### Animals

Adult C57BL/6 mice (*n* = 3 for each treatment group) were obtained from Jackson Laboratories (Bar Harbor, ME). Germ free mice were obtained from the Center for Gastrointestinal Biology and Disease Gnotobiotic Animal Core at NCSU. As previously reported, 8–12 week old mice were given a single intraperitoneal injection of doxorubicin HCl (20 mg/kg body weight, Actavis Pharma, Parsippany, NJ) to induce acute jejunal damage^2^. Animals were sacrificed 24 h after DXR treatment by first being anesthetized by isoflurane and euthanized by cervical dislocation. Control mice did not receive any DXR as a previous publication has shown no difference between saline injected and non-injected controls^1^. The small intestine was removed and the jejunum isolated and flushed with 5 mL of ice-cold 1 × PBS (Gibco, Waltham, MA) as described previously^3^. The tissue was apportioned for histologic assessment and for crypt extraction. All experimental procedures were approved by the Institutional Animal Care and Use Committee of NC State University and carried out in accordance with their guidelines.

### Cell culture

T84 cells (ATCC #CCL-248) were used within ten passages relative to initial cryotube thawing. Cells were seeded at 1 × 10^4^ cells/well (growth area 1.12 cm^2^). Cells were grown with DMEM/F12 supplemented with 2 mM l-glutamine, 5% FBS, and 1X antibiotic–antimycotic (Gibco). Cells were tested for mycoplasma contamination using the LookOut Mycoplasma PCR Detection Kit (Millipore Sigma, Burlington, MA) at the last passage.

T84 cells were plated onto transwells (Corning, 3460) to allow for polarization and grown until confluency as verified by measuring the transepithelial resistance (TER) using Millicell ERS-2 (World Precision Instruments). Once the TER was above 800 Ω cm^2^, the monolayer was assessed by brightfield microscopy. The wells were considered confluent when an intact and continuous monolayer was observed. T84 cells were exposed to DXR basally (40 µg/mL) for 9 h and then the cells were washed 3 times with 1 × PBS prior to applying new media. FITC-Dextrans (4 kDa: 46944, 10 kDa: FD10S, and 20 kDa: FD20S; Millipore Sigma, Burlington, MA) were added apically at 1 mg/mL and allowed to flux for 15 h (24 h after DXR exposure). The basal side was sampled at 9 h and 15 h after addition of the FITC-Dextran to assess flux over time. For quantification, a standard curve was made by a serial dilution. The serial dilution, blanks, and samples from the basal chamber at 24 h post DXR were measured using a Synergy 2 microplate reader (Biotek, Winooski, VT). Fluorescence was measured at excitation of 498 nm and emission at 525 nm.

### RNA isolation and RT-qPCR

#### T84 cells

Cells were lysed directly on transwells at 0, 9, and 24 h after DXR exposure with associated controls using the PureLink RNA kit (1218301, Ambion, Austin, TX) according to manufacturer specifications. Isolated RNA was assessed for purity and quantity by spectrophotometric analysis using a NanoDrop spectrophotometer (ND-2000, Thermo Scientific, Waltham, MA). cDNA from RNA using the High-Capacity cDNA Reverse Transcription Kit according to manufacturer specifications (4368814, Applied Biosystems, Foster City, CA). Gene expression was assessed using TaqMan Gene Expression Assays (Applied Biosystems): *CLDN1* (Hs00221623_m1), *CLDN2* (Hs01568822_m1), *CLDN4* (Hs00533616_s1), *OCC* (Hs00170162_m1), *ZO-1* (Hs01551861_m1), *Tric* (Tricellulin, MARVELD2, Hs00930634_m1) and *ACTB* (Hs01060665_g1).

#### Mouse jejunal crypts

Crypts were isolated as previously described^41^. Crypts were homogenized using a FastPrep-24 homogenizer (MP Biomedicals) and RNA was extracted using the PureLink RNA kit (Ambion) according to manufacturer specifications. cDNA was made using the High-Capacity cDNA Reverse Transcription kit (Applied Biosystems) according to manufacturer specifications, and gene expression was assessed using TaqMan Gene Expression Assays (Applied Biosystems): *Cldn1* (Mm00516701_m1), *Cldn2* (Mm00516703_s1), *Cldn4* (Mm00515514_s1), *Occ* (Mm00500912_m1), *Zo-1* (Mm00493699_m1), *Tric (*Tricellulin, MARVELD2, Mm01282909_m1) and *Actb* (Mm04394036_g1).

### Immunostaining

#### Collection of samples

Isolated jejunal tissues were directly embedded in OCT or placed in 4% PFA for 18 h followed by serial sucrose dehydration prior to embedding in OCT. 5 µm cryosections were cut to assess the presence of tight junctional proteins.

T84 cells were washed and fixed with methanol at − 20 °C overnight. Transwells were immersed in dry ice chilled acetone for 1 min and then promptly dried.

#### Immunofluorescence

To stain the tight junctional proteins on cryosections or transwells, sections were blocked with 3% BSA in PBS (1 h × RT) and the following primary antibodies were applied (overnight × 4 °C): mouse anti-Claudin-1 1:100 (13255S, Invitrogen); rabbit anti-Claudin-2 1:200 (51-6100, Invitrogen); rabbit anti-ZO-1 1:250 (61-7300, Invitrogen); rabbit anti-Claudin-4 1:100 (36-4800, Invitrogen); and mouse anti-Occludin 1:150 (33-1500, Invitrogen). The following secondary antibodies were applied for 1 h at room temperature: donkey anti-rabbit Alexa Fluor 488 1:250 (A21206, Invitrogen); and donkey anti-mouse Alexa Fluor 555 1:250 (A31570, Invitrogen). Sections were mounted with DAPI Hardmount (Vector Laboratories, Burlingame, CA) and imaged with an inverted Olympus IX87 fluorescent microscope using Olympus cellSens Imaging Software. Isotype control sections were imaged to monitor for non-specific staining.

### Ussing chamber ex vivo model

Whole jejunum isolated from control and DXR treated mice, full thickness pieces were mounted on 0.12 cm^2^ aperture (4 mm diameter) Ussing chambers, and the tissue was bathed in oxygenated murine Ringers solution (NaCl 109.8 mM, KCl 5.3 mM, CaCl_2_ 1.2 mM, MgCl_2_ 1.2 mM, NaHCO_3_ 25 mM, Na_2_HPO_4_ 2.4 mM, and NaH_2_PO_4_ 0.4 mM in deionized water). Tissues were oxygenated by bubbling carbogen (5% CO_2_/95% O_2_) through the Ringers solution. Tissue segments were randomly assigned to Ussing chambers and biological replicates were mounted to eliminate instrument variability as previously described^42,43^. FITC-dextrans (4 kDa, and 10 kDa) were added to the mucosal side of the chamber at a starting concentration of 1 mg/mL. Both the mucosal and serosal side were sampled after 1 h of flux. Samples were compared to a standard curve from serial dilutions of the stock solution.

### Isolation and quantification of bacterial DNA

Small intestinal mesentery, spleens, and jejunal tissue were removed and homogenized. Total DNA was extracted from homogenate using the QIAmp DNA Mini kit as per manufacturer instructions (51504 Qiagen, Hilden, Germany). 16s rDNA primers (UniF340 5′-ACTCCTACGGGAGGCAGCAG-3′ and UniR514 5′-ATTACCGCGGCTGCTGG-3′) were used and normalized to host DNA by using the primers of a single copy mouse gene, TNFα, 5′-GGCTTTCCGAATTCACTGGAG-3′ and 5′-CCCCGGCCTTCCAAATAAA-3′ as previously described^19,44^.

### 16S fluorescence in situ hybridization

#### Collection of samples

As we were interested in specifically determining if there were increases in adherent bacteria or in bacterial invasion, jejunal flushes were performed with ice cold 1 × PBS to remove non-adherent bacteria and ingesta. Flushed jejunum was fixed in 10% Zinc Formalin, dehydrated in 70% ethanol, and embedded in paraffin. 5 μm sections were applied to SuperFrost slides for FISH.

#### Oligonucleotide probes

Oligonucleotide probes for EUB338 and nonsense Non338 were synthesized with an Alexa Fluor555 fluorescent dye at the 5′ end (Thermo Fisher). The universal bacterial probe EUB338 targets most relevant bacteria. The nonsense probe Non338 was applied in parallel to check for nonspecific binding of oligonucleotide probes.

#### Hybridization

Fluorescence in situ hybridization (FISH) was performed as previously described^45^. After deparaffinization, the slides were permeabilized with lysozyme (20,000 mg/mL) in permeabilization buffer (100 mM Tris–HCl, pH 8.5) for 15 min at room temperature to improve detection of gram-positive bacteria. Hybridization was performed at 50 °C overnight in a humidified slide chamber (ACDBio) in hybridization buffer (0.9 M NaCl, 20 mM Tris–HCl, 0.1% sodium dodecyl sulfate) with 35% formamide, containing 5 ng/μL of labeled probe. The slides were then briefly washed with warmed wash buffer (100 mM Tris HCl, 0.9 M NaCl, pH 7.5) and mounted with DAPI Fluoromount (Southern Biotech).

#### Fluorescence *in-situ* hybridization imaging

Slides were imaged on an inverted Olympus IX83 microscope at 40× magnification using Olympus cellSens Imaging Software. At least 10 fields of villi and 10 fields of crypts per specimen were imaged for DAPI, GFP, and Cy3.5. Positive hybridization was identified as shapes that were consistent with bacteria (e.g. cocci, coccobacilli, or rod shaped) that were positively labeled in the Cy3.5 channel but not in the GFP channel. In addition, these were confirmed by positive staining with DAPI. A positive control was obtained from the unflushed jejunum of a mouse that was severely affected by doxorubicin and exhibited bacterial translocation.

### Statistical analysis

All quantitative results are presented as means ± SE. Data were subjected to Student’s *t* test or 2-way ANOVA with Tukey’s HSD post hoc test where appropriate, *p < 0.05, **p < 0.01, ***p < 0.001, ****p < 0.0001. Statistical analysis was performed using Prism 8 Statistical Software (GraphPad, San Diego, CA).
